# Advanced Imaging of Biochemical Recurrent Prostate Cancer With PET, MRI, and Radiomics

**DOI:** 10.3389/fonc.2020.01359

**Published:** 2020-08-19

**Authors:** Faiq Shaikh, Diana Dupont-Roettger, Jamshid Dehmeshki, Olga Kubassova, Mohammed I. Quraishi

**Affiliations:** ^1^Image Analysis Group, Philadelphia, PA, United States; ^2^Faculty of Science, Engineering and Computing, Kingston University, Kingston-upon-Thames, United Kingdom; ^3^Department of Radiology, University of Tennessee Medical Center, Knoxville, TN, United States

**Keywords:** PET, MRI, recurrent, prostate, cancer, biochemical

## Introduction

Prostate cancer is a challenging disease for both physicians and patients. It requires a multidisciplinary team of urologists, medical oncologists, radiation oncologists, radiologists, and pathologists. Current management options include radical prostatectomy (RP), external beam therapy, brachytherapy, high-intensity focused ultrasound, cryotherapy, or watchful waiting (1). Although initial management of prostate cancer is difficult, there is even more uncertainty when patients have biochemical recurrence (BCR) prostate cancer (BCRPCa), which is described as a rise in prostate-specific antigen (PSA) levels in patients with prostate cancer who have undergone surgery or radiation (1). This is because with BCRPCa, the site of recurrence can be elusive. The multidisciplinary team needs the best data possible to ascertain treatment and management options, while the patient deserves answers on the state of his disease.

After radical prostatectomy, up to a third of patients will experience BCRPCa (1). BCRPCa has risen in recent years and now affects, by some estimates, 25,000 men annually in the United States (2). Spratt et al. (2) reason that this rise is largely due to the discouragement of routine PSA screening from the US Preventative Task Force, causing an increase of men presenting with high-risk localized cancer (2, 3). This trend has also been observed in Europe and was the impetus for the European Association of Urology (EAU) latest policy statement to reevaluate PSA screening (4, 5). In addition, there is <10% utilization of adjuvant radiation therapy despite support from the American Urological Association (AUA), American Society for Radiation Oncology (ASRO), and American Society of Clinical Oncology (ASCO) (2).

The definition of BCRPCa depends on the initial treatment strategy. Any strategy that does not remove all prostate epithelial tissue will demonstrate a nadir in PSA values instead of the expected undetectable PSA values seen with RP. The AUA as well as the EAU guidelines define BCR after RP as an initial PSA value of ≥0.2 ng/ml confirmed by subsequent PSA value of ≥0.2 ng/ml (1). To predict the probability of metastasis, BCR must be taken with clinical factors such as initial PSA level, Gleason score, pathological findings after surgery, and post-BCRPCa PSA kinetics.

After confirmation of BCRPCa, imaging is vital to supply the data needed by the multidisciplinary team to direct management. Imaging can change management in up to 70% of patients (1, 6). The determination of local salvage therapy, systemic therapy, surveillance, or the addition of androgen deprivation depends on confident detection (or the lack thereof) of recurrence and distinguishing between local recurrent and metastatic disease (7). It should be noted that a change in management does not necessarily translate to a change in morbidity or mortality. Current National Comprehensive Cancer Network (NCCN) guidelines allow consideration of a multitude of imaging modalities (8). However, it is our opinion that the recommendations should be streamlined to the most effective imaging modalities available in answering the clinical question with the highest level of confidence available. The imaging studies with the highest positive rate at the lowest PSA can lead to early salvage radiation therapy.

## Current Landscape of Imaging in Biochemical Recurrence Prostate Cancer

Transrectal ultrasound (TRUS) can only evaluate the prostate bed and detects <50% of recurrence when PSA is <0.5 ng/ml (1). Computed tomography (CT) has poor anatomical resolution in the treated prostate bed, and unless recurrence is of substantial size, it is of limited use for local recurrence. CT can be helpful in evaluating for distant metastasis; however, CT has been reported to be positive in only 14% of cases (9). Any lesion seen on Tc-99m methyldiphosphonate (MDP) bone scintigraphy is highly non-specific. In fact, bone scintigraphy with BCRPCa has a positive rate of <5% when PSA is <7.0 ng/ml (10). The other obvious limitation of bone scintigraphy is that it cannot detect soft tissue recurrence.

The benefit of PET/CT is that it combines functional data ascertained by the radiotracer with limited anatomical data from the CT portion. 18F-NaF PET/CT is a bone imaging study that detects areas of increased bone turnover similar to Tc-99m MDP, allowing it to detect osseous metastases (11). Although 18F-NaF PET/CT has been shown by Jadvar et al. (12) to outperform 18-FDG PET/CT in the detection of occult osseous metastases, it has a similar constraint as bone scintigraphy in that it is confined to detecting osseous recurrence where other modalities can detect both osseous and soft tissue recurrence. The true-positive detection rate for occult osseous metastases by 18F-NaF PET/CT is 16.2%, and the median PSA levels for positive vs. negative PET/CT scans is reported as 4.4 and 2.9 ng/ml, respectively (12). 18F-FDG PET/CT, making use of glucose metabolism with a radiolabeled glucose analog, has a low sensitivity for BCRPCa, with only 28% detection of recurrence when PSA is <1.5 ng/ml (1). 11C-choline leverages the function of choline in cell membranes and lipid biosynthesis. 18F- or 11C-choline PET/CT is only of utility when PSA is >2.0 ng/ml (1). It has been observed that when PSA is <0.4 ng/ml, 11C-choline PET shows a dismal positive rate of only 21% (2). 18F-fluciclovine is a leucine amino acid analog and a novel PET radiotracer recently Food and Drug Administration (FDA) approved for use. Prostate cancer upregulates amino acid metabolism, giving 18F-fluciclovine its effectiveness as a radiotracer. At low PSA levels, it has a substantial positive detection rate. At PSA values of <1.0 ng/ml, 1.0–2.0 ng/ml, and ≥2.0 ng/ml, detection rates are reported as 72.0, 83.3, and 100%, respectively (13). Additionally, Lovec et al. (14) reported a positive rate above 50% with men with PSA values below or equal to 0.3 ng/ml. Although the NCCN guidelines report only a marginally better sensitivity and specificity range for 18F-fluciclovine compared to 11C-choline, studies comparing them head-to-head have shown that 18F-fluciclovine is superior (8, 15). Furthermore, Nanni et al. (15) reported the true positives at all PSA levels were generally higher with 18F-fluciclovine than 11C-choline.

Multi-parametric magnetic resonance imaging (mpMRI) is highly sensitive for local recurrence with its superior anatomic and tissue resolution. A positive rate of up to 94% has been reported with median PSA of 0.59 ng/ml (1). With respect to its application in prostate cancer imaging, mpMRI sequences involve various advanced sequences. The two most important sequences include diffusion-weighted imaging (DWI), which measures Brownian motion of water molecules within a voxel of tissue, and dynamic contrast enhancement (DCE) T1 imaging, which highlights vascular perfusion to tissue. DWI signal may be degraded secondary to the blooming artifact caused by surgical metallic clips or retained rectal air (16). Additionally, with short tau inversion recovery (STIR) imaging and DCE T1 imaging, osseous lesions are readily detected. In fact, MRI can detect changes in bone marrow prior to osteoblastic response which is needed for other types of bone-specific imaging (17). Post-therapy scar and fibrosis either does not enhance or demonstrates late enhancement. Malignancy, however, demonstrates early enhancement (18). The added benefit of mpMRI is that it can tease out local disease from focal treatment change that often occurs from focal therapies such as cryoablation and high-intensity focused ultrasound (18). Diagnostic CT or the CT portion of a PET/CT cannot provide the same level of anatomical detail of the treatment-altered prostate bed as mpMRI of the prostate.

**Figure 1:**
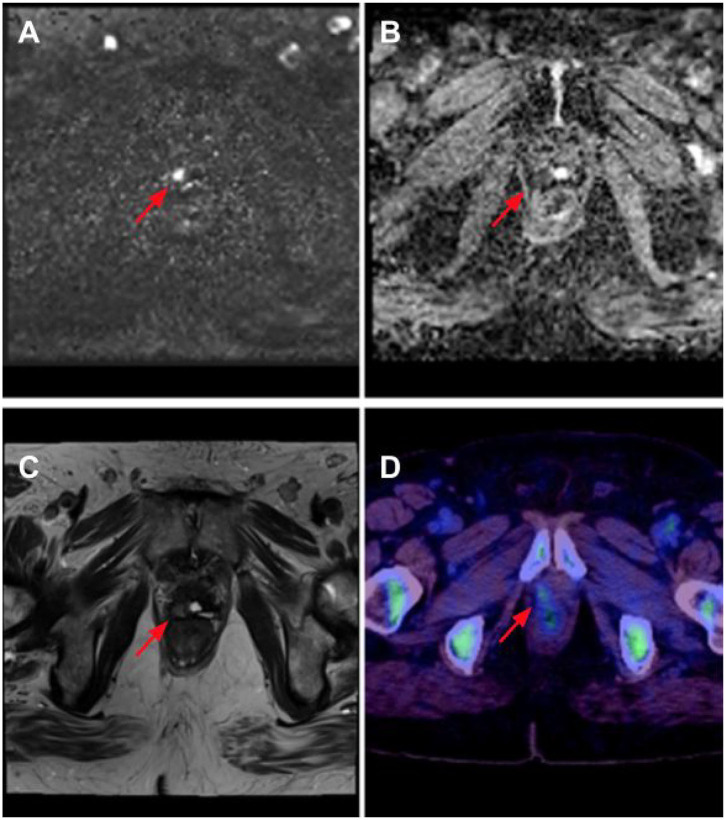
Right anterior prostate bed recurrence as seen on multi-parametric MRI (mpMRI) with 18F-fluciclovine PET/CT. There is diffusion signal on calculated b-1400 diffusion-weighted imaging (DWI) (red arrow) **(A)** with corresponding low apparent diffusion coefficient (ADC) values (red arrow). **(B)** Anatomical correlation is noted on T2 Half-Fourier acquisition single-shot turbo spin echo (HASTE) imaging (red arrow). **(C)** Lesion is confirmed to contain upregulated amino acid transport, seen in prostate cancer, in the 18F-fluciclovine image (red arrow) **(D)**.

In patients with BCRPCa, it is imperative to deliver salvage radiation therapy (RT) as early as possible (ideally PSA <0.5 ng/ml). This means that finding recurrence with the lowest possible PSA is invaluable. Of the imaging modalities available, the ones that detect disease with the lowest PSA value are 18F-fluciclovine PET/CT and mpMRI. 18F-fluciclovine is effective in detecting both local recurrence and distant metastatic disease, while mpMRI has very high utility in detecting local recurrence. In fact, a whole-body MRI would obviate the need for bone-specific imaging modalities given its superiority to both bone scintigraphy and 18F-NaF PET/CT (17). Hence, it is our opinion that there is no need for any other imaging modality except 18F-fluciclovine PET/CT combined with mpMRI, including a whole-body sequence, for BCRPCa, and ideally, 18F-fluciclovine PET/MRI, if available, for the added benefit of superior osseous detection (Figure 1). This approach will give the multidisciplinary team the structural and functional information to make early management decisions with high confidence.

## Future Directions in Biochemical Recurrence Prostate Cancer Imaging

Molecular imaging approaches applied in the management of BCRPCa management include prostate-specific membrane antigen (PSMA) radiotracers bound either to gallium (68Ga-PSMA) or to fluoride (18F-DCFPyL). PSMA is a membrane glycoprotein that is overexpressed by prostate cancer cells. Ga-PSMA PET is currently undergoing Phase III trials in the US and appears to outperform 18F-fluciclovine with a positive rate of 73% at a PSA range as low as 0.5 to 1.0 ng/ml and a positive rate of >50% at the remarkably low PSA range of 0.20–0.29 ng/ml (1). It should be noted that 68Ga-PSMA is already clinically available in Europe and outperforms 18F-fluciclovine (19). 18F-DCFPy is a PSMA radiotracer that produces images with higher resolution and is currently in phase II trials (2). It has been shown to successfully identify recurrent disease and lead to a change in management in 60% of patients and in up to 28% of patients who had negative CT or MR findings (20). It has been shown to detect bone metastases as accurately as 18F-NaF PET/CT but is superior to the latter given its ability to detect non-osseous disease at low PSA values, making it a more useful study overall (21).

**Figure 2:**
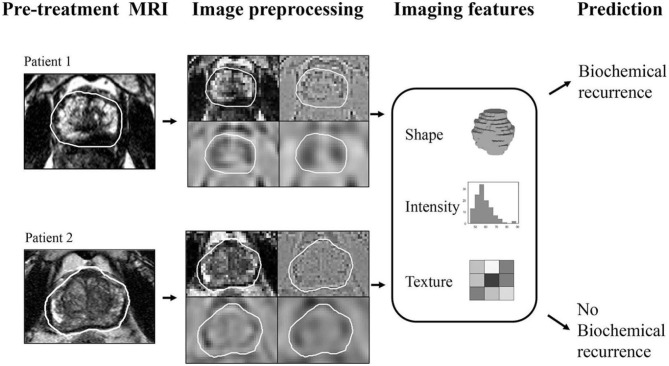
Graphical schema of the radiomics process that involves lesion identification, drawing regions of interest, image preprocessing followed by radiomic feature extraction and classification that provides the imaging biomarker for predicting biochemical recurrence. Reused from Fernandes et al. (28) under the Creative Commons License.

BCRPCa as well as primary prostate cancer is ripe for quantitative imaging biomarker development using radiomics as a methodology. Radiomics may be defined as a process of extracting quantified data from medical images as single-order (histogram-based) and second-order (texture analysis-based) features, which are then classified into clusters (or signatures) that best align with an underlying pathophysiologic process (Figure 2). Radiomic analysis performed on pretreatment mpMRI has been shown to predict BCRPCa, which has implications for predicting response to adjuvant therapy (22, 23). In addition, radiomic texture analysis has been shown to predict biochemical relapse as well as BCRPCa-free survival after prostatectomy [area under the curve (AUC) 0.76] (24). Furthermore, MR radiomic signatures [using T2W and apparent diffusion coefficient (ADC) images] can accurately predict the response to carbon ion radiotherapy (CIRT) for prostate cancer as well (25). Recently, radiomics has been shown to predict Decipher score (an mRNA-based genomic test that predicts the occurrence of prostate cancer metastasis after radical prostatectomy) by differentiating between low and intermediate/high scores (with an AUC of 0.92) (26, 27).

Imaging is central to BCRPCa treatment decisions. Current practice in the US should be reformed to use 18F-fluciclovine and moving to a PSMA-based radiotracer as currently approved in Europe once FDA approved in the USA in conjunction with mpMRI or as PET/MR where available. The future is bright in the fight against BCRPCa with growing research in imaging-based precision medicine practices including radiomics-based imaging biomarkers.

## Author Contributions

FS contributed to manuscript writing, focusing on PET/CT and radiomics. DD-R, JD, and OK contributed to manuscript writing and provided technical input. MQ contributed to manuscript writing focusing on clinical, PET/CT, and MRI. All authors contributed to the article and approved the submitted version.

## Conflict of Interest

FS, DD-R, JD, and OK are employees of Image Analysis Group, Philadelphia, PA, USA. The remaining author declares that the research was conducted in the absence of any commercial or financial relationships that could be construed as a potential conflict of interest.
